# Optimal cut-off value for detecting colorectal cancer with fecal immunochemical tests according to age and sex

**DOI:** 10.1371/journal.pone.0254021

**Published:** 2021-07-16

**Authors:** Mercedes Vanaclocha-Espi, Josefa Ibáñez, Ana Molina-Barceló, María José Valverde-Roig, Andreu Nolasco, Francisco Pérez-Riquelme, Mariola de la Vega, Isabel Portillo, Dolores Salas

**Affiliations:** 1 Foundation for the Promotion of Health and Biomedical Research-Public Health Research FISABIO–Public Health Research, Valencia, Spain; 2 General Directorate Public Health, Valencian Community, Spain; 3 University of Alicante, Alicante, Spain; 4 General Directorate of Public Health, Murcia Region, Spain; 5 Biomedical Research Institute of Murcia (IMIB-Arrixaca-UMU), University Hospital “Virgen de la Arrixaca”, University of Murcia, Murcia, Spain; 6 General Directorate of Assistance Programs, Canary Islands, Spain; 7 The Basque Health Service, Basque Country, Spain; University of Munich, GERMANY

## Abstract

In the fecal immunological test, a suitable cut-off value may be selected to classify results as either positive or negative. Our aim is to estimate the optimal cut-off value for detecting colorectal cancer in different age and sex groups. This is a multicentric retrospective cohort study of participants in CRC screening programs with FIT between 2006 and 2012. A total of 545,505 participations were analyzed. Cancers diagnosed outside of the program were identified after a negative test result (IC_test) up until 2014. The Wilcoxon test was used to compare fecal hemoglobin levels. ROC curves were used to identify the optimal cut-off value for each age and sex group. Screening program results were estimated for different cut-off values. The results show that the Hb concentration was higher in colorectal cancer (average = 179.6μg/g) vs. false positives (average = 55.2μg/g), in IC_test (average = 3.1μg/g) vs. true negatives (average = 0μg/g), and in men (average = 166.2μg/g) vs. women (average = 140.2μg/g) with colorectal cancer. The optimal cut-off values for women were 18.3μg/g (50-59y) and 14.6μg/g (60-69y), and 16.8μg/g (50-59y) and 19.9μg/g (60-69y) for men. Using different cut-off values for each age and sex group lead to a decrease in the IC_test rate compared to the 20μg/g cut-off value (from 0.40‰ to 0.37‰) and an increase in the false positive rate (from 6.45% to 6.99%). Moreover, test sensitivity improved (90.7%), especially in men and women aged 50-59y (89.4%; 90%) and women aged 60-69y (90.2%). In conclusion, the optimal cut-off value varies for different sex and age groups and the use of an optimal cut-off value for each group improves sensitivity and leads to a small decrease in IC_tests, but also to a larger increase in false positives.

## Introduction

Colorectal cancer screening programs (CRCSP) are currently the main strategy for the early detection of colorectal cancer (CRC). The purpose of these programs is to detect early-stage CRC and adenoma, which are precursor lesions of CRC. A fecal occult blood test carried out every two years, followed by a colonoscopy to confirm the diagnosis, is one of the strategies recommended in the European guidelines for quality assurance in colorectal cancer screening [1]. Also, the strategy is cost-effective compared to no screening [2].

Fecal occult blood tests measure human hemoglobin (Hb) concentration in feces. There are two methods: guaiac and immunochemical (FIT). The FIT allows for automated reading and the selection of cut-off values to classify results as positive or negative.

Screening with the guaiac test has shown to reduce CRC mortality by 15% [3], while screening with the FIT using a cut-off value of 20μg/g of feces has shown to reduce CRC by 22% compared to the non-screened population [4]. Furthermore, the FIT has proven to be more effective in detecting advanced neoplasia and is also more widely accepted by the population [5–7].

In Spain, CRCSPs depend on the healthcare services of the various regions. The FIT with a cut-off value of 20μg/g (100ng/ml) is the screening test currently used.

Population distribution by age and sex is a factor that influences the impact of CRCSPs because CRC incidence and mortality is lower in women than in men, and tends to increase with age. Worldwide cancer incidence in 2018 was estimated at 20.9 per 100,000 women and 30.3 per 100,000 men [8].

Differences in CRC incidence can be seen in screening results, specifically in the positive test rate or the positive predictive value (PPV). A study describing the results of CRCSPs in Spain found that the rate of positive tests and PPVs is higher in men than women [9]. Salas et al. (2014) showed that a lower number of lesions (adenoma, CRC) are detected in women, and particularly in the younger group of women aged 50–59 years [10]. A study showed that when a cut-off value of 10μg/g is used for the entire population, the rate of positive tests continues to be higher in men [11].

Most studies evaluating the quality of CRCSP based on FIT use a single cut-off point for the entire target population [12–16]. A study found that cut-off points above 25μg / g hardly affects the positive predictive value of the test [12]. Chen et al. (2007) found that FIT sensitivity and specificity varied for different age and sex groups with the same cut-off value, concluding that a cost-effective cut-off value was 110ng/ml [14]. One study estimates variations in program results when different cut-off values are used, showing the differences in the results by age and sex [15]. Among the studies to determine the optimal FIT cut-off value, the results of a Japanese study concluded that the optimal cut-off value was estimated at 200ng/ml [13]. Furthermore, a Spanish study estimated the optimal cut-off value for detecting CRC at 115ng/ml [16].

Studies showing that the amount of Hb in feces is different in men and women [17], in addition to the differences found in CRCSP results by age and sex, have led several authors to conclude that these two factors must be present in order to predict the probability of a lesion in the colon or rectum [18–20]. Kim et al. (2016) also suggest that factors such as smoking and exercise must be considered in addition to age and sex to determine the probability of CRC [19]. A study suggests that using risk-adapted cut-off values may help to achieve targeted levels of PPV [21]. A number of studies analyzing the results of CRCSPs that use the FIT conclude that cut-off values for this test must be personalized by age and sex to improve program results [18–22].

The purpose of this study was to identify sex- and age-specific FIT cut-off values and evaluate CRCSP results for different cut-off values and by age and sex groups, thereby providing information for CRCSPs on FIT use that is personalized in accordance with these factors. The analysis of a large population of CRCSP participants allows us to offer populational results for Spain.

## Materials and methods

The CRIBEA-CIN project is a multicentric retrospective cohort study of participants in CRCSPs carried out in the Valencian Community, Region of Murcia, Canary Islands, and the Basque Country (all located in Spain). The characteristics of these programs can be seen in the study of Vanaclocha-Espi et al., 2017 [23]. This project was born with the aim of studying the balance between predictive indicators of the benefits and adverse effects of CRCSPs.

This study was approved (May 29th, 2015) by the Clinical Research Ethics Committee (CEIC) of the General Directorate of Public Health (DGSP) and the Advanced Public Health Research Centre (CSIP) of the Valencian Community (Reference: PI15/02108). Taking into account the project design, its large sample size and that the data are anonymized, the ethics committee approved carrying out the study without requesting individualized consent from each subject, following the regulations of the Declaration of Helsinki currently in effect (October 2008, Seoul).

The project analyzed information from all participations of the cohort of men and women aged 50–69 invited to take part in the CRCSPs from 2006 until December 2012. In total, 558,629 FIT were carried out. Interval cancers were identified in this population up until 2014 through with Cancer registries and using an internationally defined protocol [24]. Identified interval cancers either had a negative FIT result the last time they participated in the program and were diagnosed within 0–24 months or longer in the event that the following invitation was delayed, or had a positive FIT result and a negative CRC result after undergoing a colonoscopy and were diagnosed in the follow-up period before the next invitation. The data were obtained from the information systems of each of the participating CRCSPs in an anonymized way and were combined in a common database.

This study analyzed data from participations with FIT and excluded any participations with an unknown value in the qualitative test result (n = 13.124). The final sample used in this study was made up of 545,505 participations with FIT.

Over the study period, the participating CRCSPs used FITs from different manufacturers. For this study, the test results were standardized to μg/g of feces in order to make them comparable.

The CRCSPs participating in this study used a cut-off value of 20μg/g of feces. An Hb concentration in feces of >20μg/g was considered a positive result requiring colonoscopy follow-up in the Valencian Community, the Region of Murcia, and the Canary Islands, while a concentration of ≥20μg/g was considered positive in the Basque Country.

**Fig 1:**
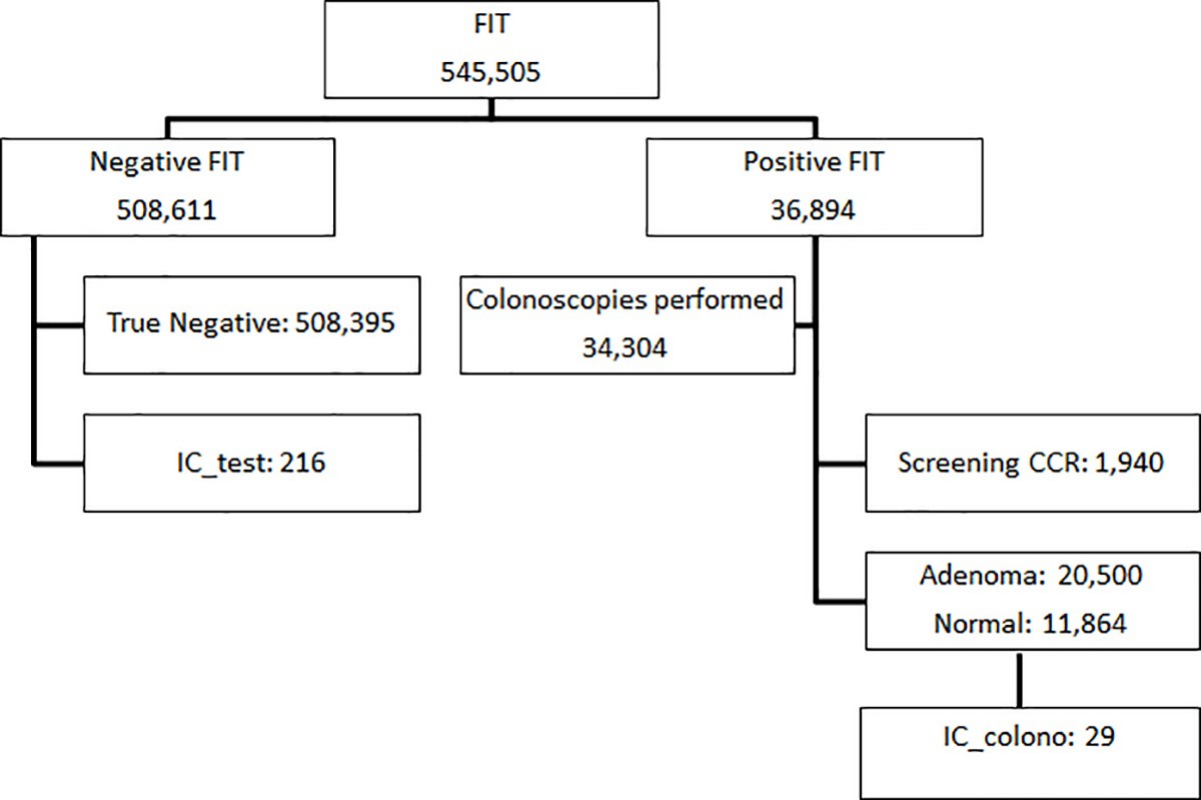
Study flow diagram. FIT, fecal immunochemical test; CRC, colorectal cancer; IC_test, test interval cancer; IC_colono, colonoscopy interval cancer.

The population that completes the screening process receives one of 5 possible results (Fig 1):

- True negative (TN): patients with a negative FIT result who do not have a diagnosis of CRC in the period between two invitations.
- False negative or FIT interval cancer (IC_test): patients diagnosed with CRC, although the FIT gave a negative result. These cases of CRC are diagnosed after a negative screening process and before the following invitation.
- True positive or screening cancer (SC): patients that have CRC and got a positive FIT result.
- False Positive (FP): patients that through colonoscopy are confirmed to not have CRC after a positive FIT result. Adenomas are included in this group. Patients with Adenomas detected are included in colonoscopy surveillance every 3 or 5 years. And the rest of the patients are incorporated into biannual FIT screening after 5 years.
- Colonoscopy IC (CI_colono): patients that got a positive FIT result and a normal result or an adenoma diagnosis in the colonoscopy, but are diagnosed with CRC before the next program test.

### Statistical analysis

A descriptive analysis was carried out using boxplots of Hb concentration in feces from negative and positive FIT results, as classified by the CRCSPs. The non-parametric Wilcoxon test was used to compare the distribution of Hb levels in feces between TN and IC_test for negative FIT results, compare CRC and FP for positive FIT results, and compare the CRC and IC_test rate between women and men. The Spearman correlation test was applied to assess the impact of age on the quantity of Hb in feces.

ROC curve analyses were carried out to detect the diagnostic accuracy of the FIT and the cut-off value that maximizes sensitivity and specificity for CRC detection. The discriminatory ability of the test was assessed by estimating the area under the curve (AUC). ROC curve analyses were carried out for both the total sample and stratified by age and sex groups: women aged 50–59, women aged 60–69, men aged 50–59, and men aged 60–69.

Finally, the results of the screening process were simulated for different cut-off values, in particular for different optimal cut-off values for each age and sex group determined by means of a ROC curve analysis (O1); for an optimal cut-off value for the total sample determined by means of a ROC curve analysis (O2); and for cut-off values of 20, 25, 30, 35, and 45μg/g of feces. The validity of the FIT was analyzed for each of these cut-off values by calculating the sensitivity and specificity. Furthermore, the FP rate, IC_test rate, SC rate, and number of colonoscopies recommended were estimated for each of the cut-off values under study. The rates at the different cut-off points were compared with the rates at the 20 μg/g cut-off using a Chi-square analysis. The results were simulated both for the total sample and by age and sex group. The statistical analyses were carried out using the statistical program R.

## Results

**Fig 2:**
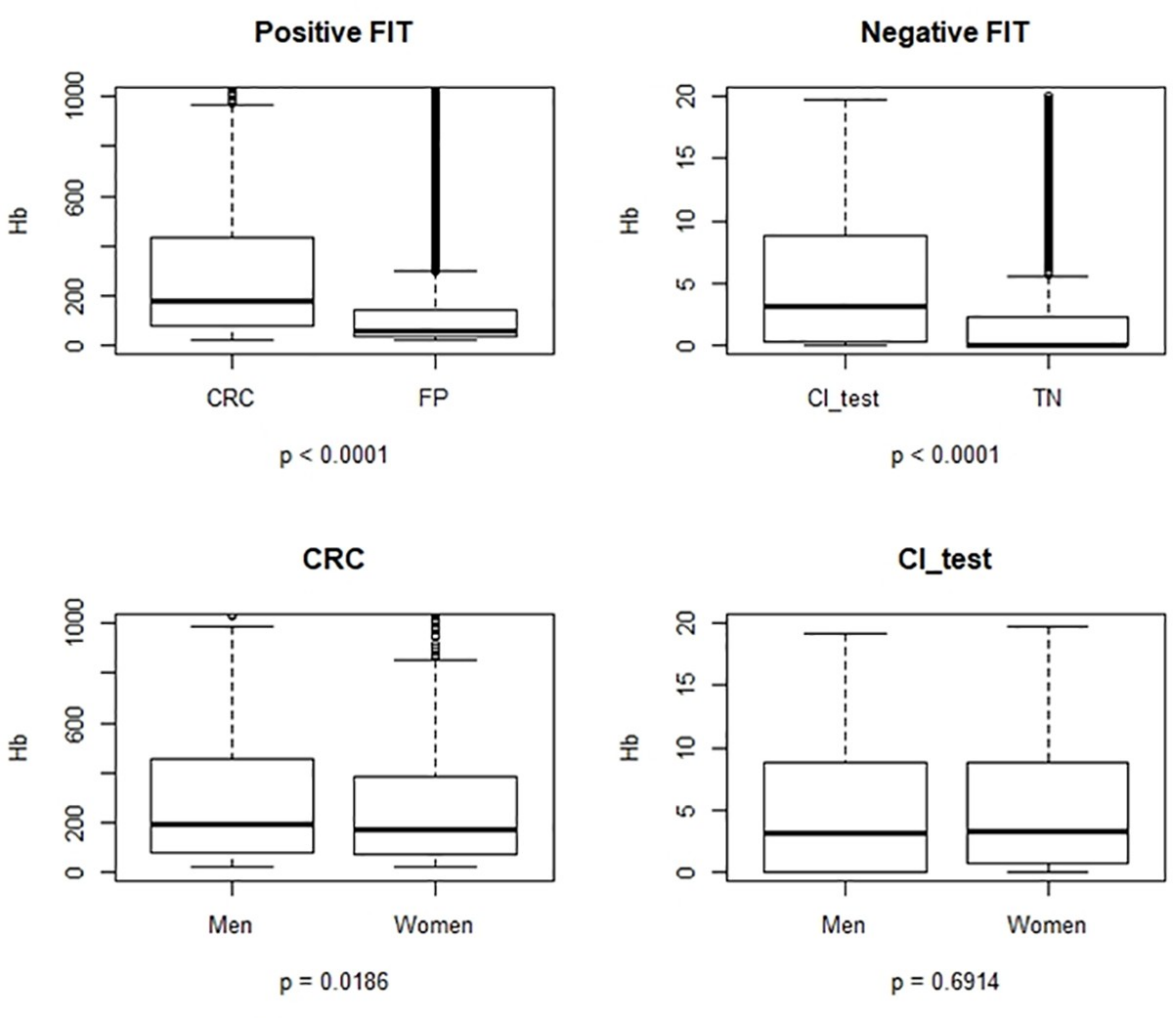
Distribution of fecal Hb levels according to the results of the screening process. FIT, fecal immunochemical test; CRC, colorectal cancer; FP, false positive; TN, true negative; IC_test, test interval cancer.

Fig 2 shows the differences in fecal Hb concentration between CRC and FP, both of which are positive FIT results (p<0.001). Differences in fecal Hb concentration between TN and IC_test, both of which are negative FIT results, can also be seen. Hb levels were higher in men with CRC (average = 166.2μg/g) than women with CRC (average = 140.2μg/g), P = 0.0186. No differences were found between men and women with IC_test (p = 0.6914). A slight positive correlation was found between age and fecal Hb concentration (Spearman correlation = 0.01, p<0.001). This is not shown in the tables and figures.

**Fig 3:**
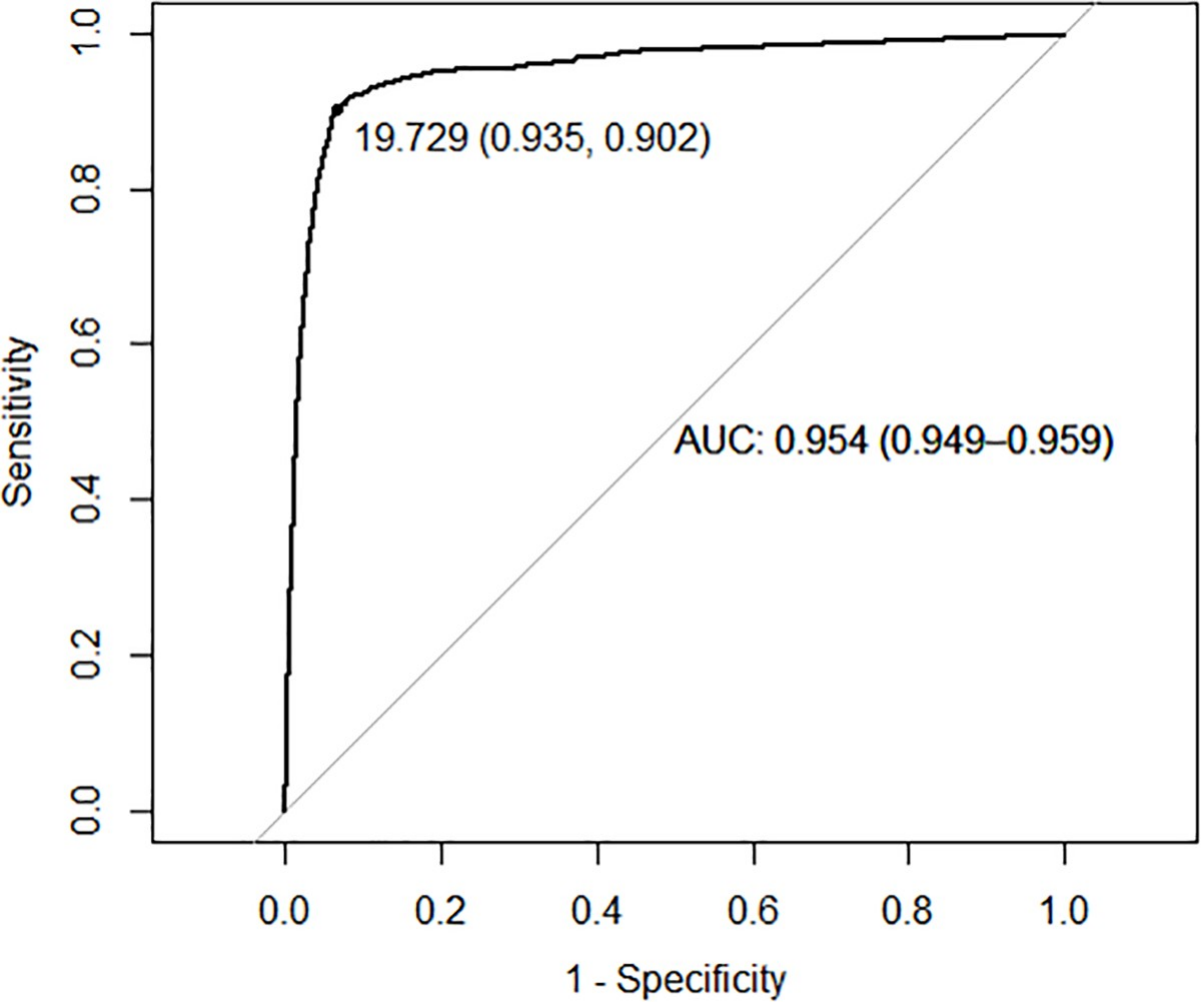
ROC curve for the total sample, optimal cut-off value for CRC diagnosis. AUC, area under the curve.

The ROC curve analysis estimated an optimal cut-off value (O2) of 19.73μg/g of feces for the total sample. This value determined the highest combination of sensitivity (90.2%) and specificity (93.5%) for CRC diagnosis. The AUC was 95.4% (94.9%-95.9%), meaning that the FIT is highly capable of correctly classifying patients with CRC and people that do not have this cancer (Fig 3).

**Fig 4:**
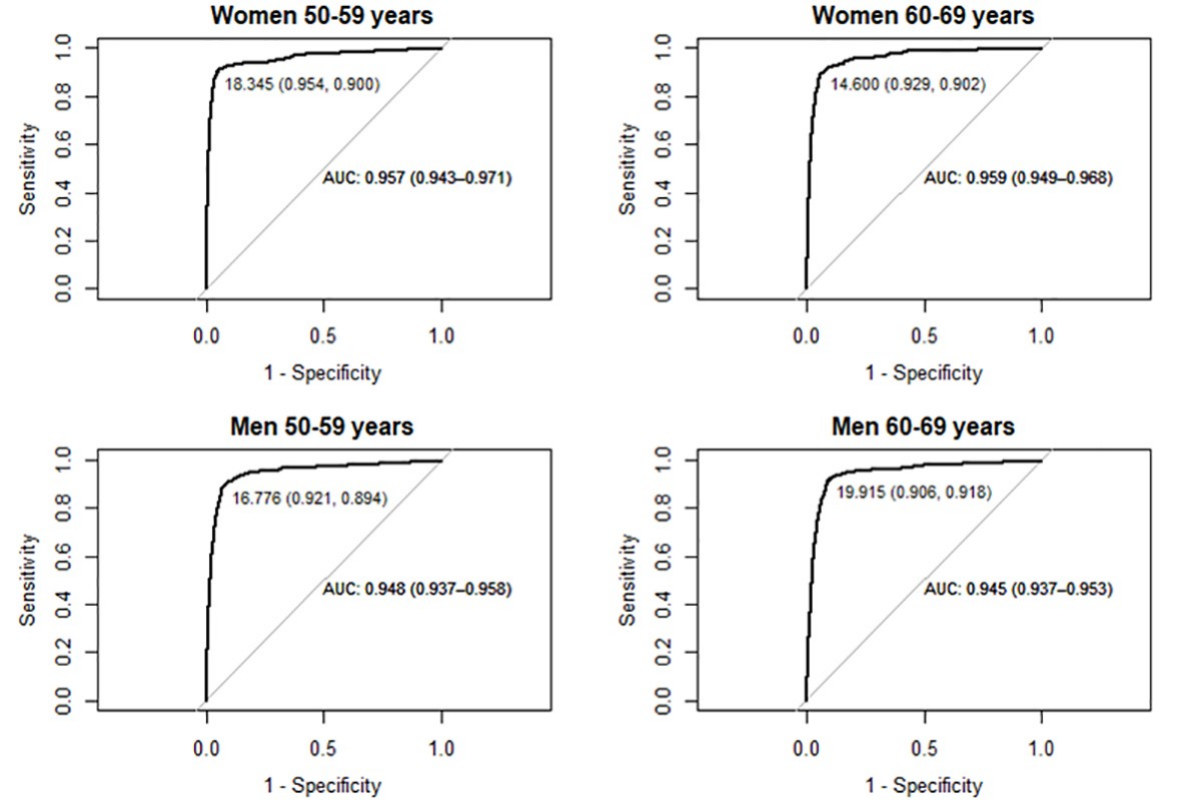
ROC curves by age and sex group, optimal cut-off values for CRC diagnosis. AUC, area under the curve.

ROC curves were estimated by age and sex group in order to determine a specific optimal cut-off value for CRC detection (O1), and this was set at 18.35μg/g for women aged 50–59; at 14.60μg/g for women aged 60–69; at 16.8μg/g for men aged 50–59; and at 19.92μg/g for men aged 60–69. The AUC of all the estimated cut-off values was close to 100% and with confidence intervals of between 93.7% and 97.1%. Therefore, the FIT is highly capable of correctly classifying participants in all the age and sex groups analyzed (Fig 4).

Table 1 shows the simulations of general CRCSP results for different FIT cut-off values. A very similar sensitivity would be obtained for the cut-off values O1, O2, and 20μg/g, and the sensitivity falls from 90.11% to 79.5% if the cut-off value increases from 20 to 40μg/g. As compared to the cut-off value of 20μg/g, with cut-off value O1 there is an increase in the FP (0.54%) and SC (0.02‰) rates, and a decrease in the IC_test rate (0.03‰).

**Table 1:** Results of the screening process (rates, sensitivity, specificity) and change in rates vs cut-off 20μg/g.

Cut-off value μg/g of feces		CRC		Sensitivity		Specificity		PPV		FP		IC_test		SC	
		Yes	No	%	95%CI	%	95%CI	%	95%CI	Rate %	change vs. 20	Rate ‰	change vs. 20	Rate ‰	change vs. 20
O1	Positive	1,981	38,132	90.7	(89.44–91.88)	93.0	(92.91–93.05)	4.9	(4.73–5.15)	6.99	0.54*	0.37	-0.03	3.63	0.02
	Negative	204	505,190												
O2	Positive	1,970	35,365	90.2	(88.91–91.41)	93.5	(93.43–93.56)	5.3	(5.05–5.50)	6.48	0.03	0.39	-0.01	3.61	0
	Negative	215	507,957												
20	Positive	1,969	35,211	90.1	(88.86–91.37)	93.5	(93.45–93.58)	5.3	(5.07–5.52)	6.45		0.40		3.61	
	Negative	216	508,109												
25	Positive	1,881	29,335	86.1	(84.64–87.54)	94.6	(94.54–94.66)	6.0	(5.76–6.29)	5.38	-1.07*	0.56	0.16*	3.45	-0.16
	Negative	304	513,985												
30	Positive	1,828	26,037	83.7	(82.11–85.21)	95.2	(95.15–95.26)	6.6	(6.27–6.85)	4.77	-1.68*	0.65	0.25*	3.35	-0.26*
	Negative	357	517,283												
35	Positive	1,789	23,524	81.9	(80.26–83.49)	95.7	(95.62–95.72)	7.1	(6.75–7.38)	4.31	-2.14*	0.73	0.33*	3.28	-0.33*
	Negative	396	519,796												
40	Positive	1,737	21,594	79.5	(77.80–81.79)	96.0	(95.97–96.08)	7.5	(7.11–7.78)	3.96	-2.49*	0.82	0.42*	3.18	-0.43*
	Negative	448	521,726												

CRC, Colorectal cancer; PPV, positive predictive value; FP, false positive; IC_test, test interval cancer; SC, screening cancer rates; O1, different optimal cut-off value for each age and sex group, 18.35μg/g in women aged 50–59, 14.60μg/g in women aged 60–69, 16.78μg/g in men aged 50–59, and 19.91μg/g in men aged 60–69; O2, optimal cut-off value of 19.73μg/g for the total sample; CI, confidence interval.

*p_valor<0.05.

**Fig 5:**
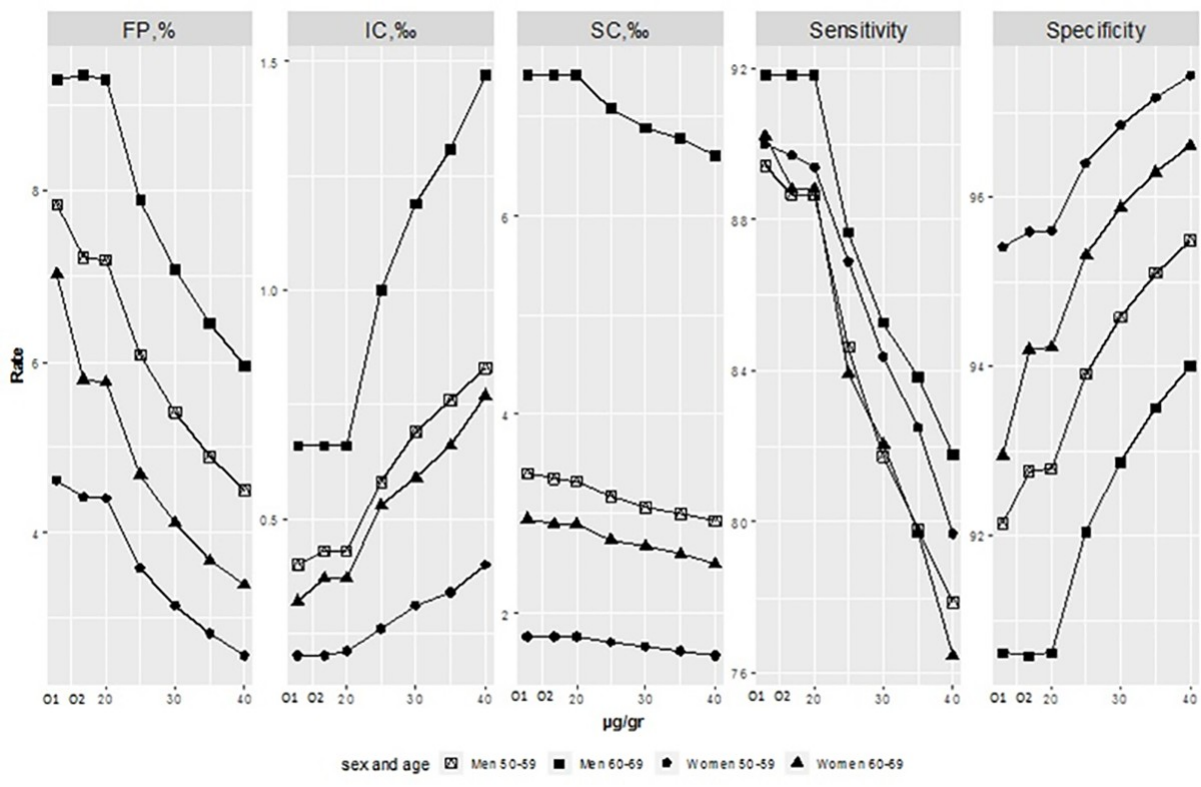
Sensitivity, specificity and rates with different FIT cut-off values for each age and sex group. FIT, fecal immunochemical test CRC; FP, false positive; IC, interval cancer; SC, screening cancer; O1, different optimal cut-off value for each age and sex group, 18.35μg/g in women aged 50–59, 14.60μg/g in women aged 60–69, 16.78μg/g in men aged 50–59, and 19.91μg/g in men aged 60–69; O2, optimal cut-off value of 19.73μg/g.

CRCSP results by age and sex and for different cut-off values are shown in Fig 5. Cut-off value O1 increased sensitivity and reduced specificity for women aged 50–59, women aged 60–69, and men aged 50–59 compared to all other cut-off values common to the entire population.

**Fig 6:**
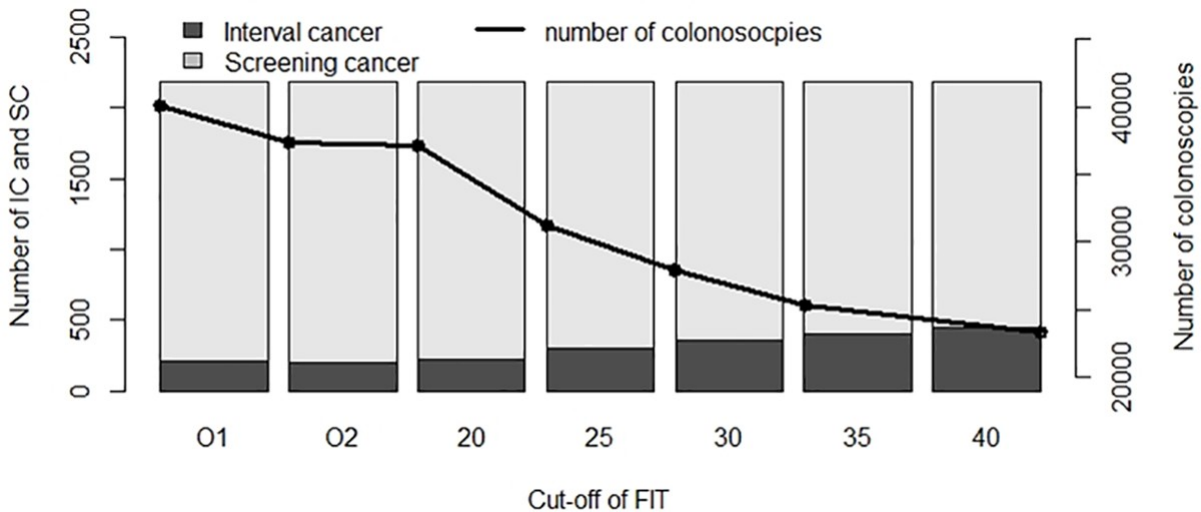
Number of colonoscopies recommended and proportion of IC and SC for different Hb cut-off values. IC, interval cancer; SC, screening cancer; O1, different optimal cut-off value for each age and sex group, 18.35μg/g in women aged 50–59, 14.60μg/g in women aged 60–69, 16.78μg/g in men aged 50–59, and 19.91μg/g in men aged 60–69; O2, optimal cut-off value of 19.73μg/g for the total sample.

Fig 6 shows that for cut-off value O1, a higher number of colonoscopies were required (40,113), and the number of IC and SC varied very little in comparison to cut-off value O2 and the regular cut-off value of 20μg/g.

## Discussion

The optimal cut-off value that maximized FIT sensitivity and specificity is different for each sex and age group. The use of an optimal cut-off value for each age and sex group enhances the benefits in terms of increased CRC detection, but also increases the adverse effects of the program.

The program results simulations are slightly different when using a cut-off value of 20μg/g for the entire population or different cut-off values for each age and sex group. The use of different cut-off values multiplies program benefits, with an increase of the SC rate and a reduction of the IC_test rate. However, it also leads to an increase in the adverse effects of the program, namely growth of the FP rate. The increase in the rate of FP leads to an increase in the number of colonoscopies. In terms of the balance between program benefits and adverse effects, the increase in the FP rate is larger than the increase in benefits. Therefore, the results of this study show that the balance is still unclear.

Grobbee et al., 2017 concluded that changing the cut-off value for each sex could even out the number of lesions not detected in screening between men and women [18]. The results of CRCSPs using different cut-off values for each age and sex group have particularly significant changes for men and women aged 50–59 and women aged 60–69: a decrease in the IC_test rate and an increase in the SC and FP rate.

For men and women aged 50–59 and women aged 60–69, the optimal cut-off value is slightly lower than the value used in conventional practice in European programs (20μg/g). On the contrary, the optimal cut-off value for men aged 60–69 is the same as the value used in conventional practice. Therefore, it would not be possible to decrease the IC_test rate in this group with the cut-off points used in the study. This study has shown that the quantity of Hb in feces is very low in IC_test, which suggests that it would be very difficult to decrease this rate even if we lowered the FIT threshold. The results suggest that the amount of Hb in feces in IC_test is undetectable when the test is performed. This could be due to the particular characteristics of these cancers, for example, the fact that IC tends to be located in the proximal colon or rectum [25].

Our study shows that men with CRC have a higher amount of Hb in feces than women with CRC. These results are in line with other investigations showing that male CRCSP participants have higher fecal Hb concentrations that female CRCSP participants [17].

In terms of sensitivity, program results using a cut-off value of 20μg/g show that the FIT is most sensitive in men aged 60–69, followed by women aged 50–59 and women aged 60–69. It is least sensitive in men aged 50–59. A meta-analysis of scientific studies evaluating FIT sensitivity by sex, found 3 studies that showed sensitivity is higher in men than women, as well as another 3 studies evaluating FIT sensitivity by age showed sensitivity is higher in the 50–59 age group than the 60–69 age group [26]. Our study, by combining age groups and sex, provides more specific results on the changes in the sensitivity and specificity of the FIT than the usual studies that classify by sex and age independently.

The use of different cut-off values for each age and sex group improves test sensitivity when CRCSP results are evaluated for women aged 50–59, women aged 60–69, and men aged 50–59. These results support the hypothesis of Alvarez-Urturi et al. (2016) that, to improve FIT performance in CRCSP, cut-off values could be personalized by age and sex [22]. We have seen that if we evaluate FIT performance in the entire population, the use of different cut-off values does not lead to significant changes; namely, sensitivity generally remains at around 90% and specificity at 93% for both criteria.

In contrast, the negative side of using different cut-off values for each age and sex group is that the number of colonoscopies recommended increases, as well as the FP rate. FP in particular entails a negative effect on CRCSPs, as people may undergo unnecessary colonoscopy (which increases the burden on healthcare workers) and suffer the stress of thinking they are ill. Furthermore, colonoscopies carry certain risks, such as potential complications of the procedure (e.g. perforation or hemorrhage), and this is an adverse effect of CRCSPs. One study associates men with a lower risk of FP [27]. Using estimated cut-off values for each age and sex group would increase the FP rate in women and also in men aged 50–59, which could influence and maximize the differences between the sexes found in this adverse effect.

The diagnosis confirmation test, i.e., the colonoscopy, is carried out by the health system of each region in Spain, in units that are not exclusive to CRCSPs. Colonoscopies resulting from CRCSPs are a significant burden for the health system and the number of people referred for this test must be well adjusted.

One limitation of the study is that it was not possible to analyze the sensitivity and specificity for both CRC detection and adenomas, as we cannot count the number of adenomas not detected by the FIT. Another limitation is that other variables that have been shown to be related to FIT performance, such as tobacco use or personal history of FIT screening tests in previous participations [19, 28] are not included in the study.

Following the recommendations of the European CRC screening guide [1], population-based programs should be organizationally adapted according to the characteristics and resources of each program. The results of this study can serve as the basis for making decisions to modify the cut-off points. In any case, following the recommendations, information should be provided to the population on both the benefits and the adverse effects of applying different cut-off points.

Our investigation provides new evidence on how to use the FIT in a personalized manner by age and sex. We have seen that using different cut-off values for different age and sex groups generally provides different CRCSP results to the conventional cut-off value in terms of sensitivity and specificity, and also increases the number of colonoscopies recommended in CRCSPs (2,933 additional colonoscopies) and the FP rate (2,921 additional FPs). Both the benefits and adverse effects of using different cut-off values for each age and sex group must be considered when deciding the cut-off value to be used.
